# Investigating Vitreous Cytokines in Choroidal Melanoma

**DOI:** 10.3390/cancers15143701

**Published:** 2023-07-21

**Authors:** Hakan Demirci, Lu Tang, F. Yesim Demirci, Cem Ozgonul, Sarah Weber, Jeffrey Sundstrom

**Affiliations:** 1 Department of Ophthalmology and Visual Sciences, University of Michigan, Ann Arbor, MI 48105, USA; 2 Department of Biostatistics, School of Public Health, University of Pittsburgh, Pittsburgh, PA 15261, USA; 3 Department of Human Genetics, School of Public Health, University of Pittsburgh, Pittsburgh, PA 15261, USA; 4 Department of Ophthalmology and Visual Sciences, Penn State University, Hershey, PA 17033, USA

**Keywords:** vitreous, cytokines, chemokines, choroidal melanoma, uveal melanoma, ocular cancer, prognosis, gene expression profiling, liquid biopsy, biomarkers

## Abstract

**Simple Summary:**

Uveal melanoma (UM) is a rare cancer highly prone to metastasis. Metastatic risk is currently predicted using clinical and/or tumor biopsy-based information. While tumor biopsy-based molecular information is helpful, there is an unmet need for a liquid biopsy-based approach as a more advantageous alternative to tumor biopsy. Given that UMs primarily arise in the posterior eye segment, vitreous (which fills the posterior eye cavity) represents a fluid source enriched in tumor-derived molecules. Inflammatory microenvironment indicates poor prognosis in UM and cytokines are key mediators of immune response/inflammation. Vitreous cytokine analysis may therefore increase our understanding of UM and inform future clinical strategies. In this study, we analyzed 41 vitreous cytokines in 32 eyes (18 with posterior UM and 14 controls) and identified 26 UM-associated and 6 prognosis-relevant cytokines. Our findings further support the value of vitreous cytokine analysis in UM and the need for additional studies to establish the best candidates for biomarker development and/or therapeutic targeting.

**Abstract:**

Due to the close relationship between the vitreous and posterior eye layers, the microenvironment of these layers can affect the composition of the vitreous. Molecular analysis of the vitreous may therefore provide important insights into the pathogenesis of chorioretinal diseases. In this study, vitreous cytokines (n = 41) were evaluated to gain further insights into the tumor microenvironment in uveal melanoma (UM) arising from the choroid (CM). Cytokine levels were measured using a bead-based multiplex immunoassay panel in vitreous samples obtained from 32 eyes, including 18 with CM and 14 controls. Median fluorescence intensity values were extracted and used as relative quantification of the cytokine abundance. Vitreous cytokine levels were compared between the CM and non-CM groups and between different prognostic categories within the CM group (classified as having low or high metastatic risk using tumor biopsy-based gene expression profiling). Correlations between vitreous cytokine levels and tumor dimensions were also evaluated. Our analysis revealed twenty-six vitreous cytokines significantly upregulated in CM-affected eyes compared to the control eyes. Within the CM group, six vitreous cytokines showed altered levels (five upregulated and one downregulated) in eyes with high- vs. low-risk tumors. Levels of these six plus several other cytokines showed correlations with the tumor dimensions. In conclusion, our study has uncovered several UM-relevant vitreous cytokines, worthy of follow-up in larger studies as potential candidates for liquid biopsy-based biomarker development and/or new therapeutic targeting.

## 1. Introduction

Vitreous humor fills the posterior eye compartment and is not only made of water (99%), hyaluronic acid, and collagen but also contains diverse proteins, metabolites, and nucleic acids [1,2]. Due to the close anatomic and functional relationship between the vitreous and posterior eye layers (retina, retinal pigment epithelium, and choroid), any change in the microenvironment of these layers may also affect the composition of the vitreous. Unlike the aqueous humor which is constantly renewed in the front part of the eye, vitreous remains more stagnant in the back of the eye, acting as a reservoir that can better reflect the chronic state of the eye [3]. Protein secretion or shedding from the posterior eye layers may significantly affect the vitreous proteome, which appears to include both unique and shared proteins compared to other body fluid proteomes [1,4]. Previous studies have demonstrated that critical posterior eye disease proteins (e.g., soluble mediators such as cytokines) can be detected in the vitreous, hence the analysis of the vitreous can shed further light on the pathogenesis of chorioretinal diseases and inform future clinical management (i.e., diagnostic, prognostic, and therapeutic) strategies [5,6,7,8].

Uveal melanoma (UM) arises from the melanocytes within the uveal tract (iris, ciliary body, and choroid) of the eye, and while rare in incidence, it represents the most common primary intraocular cancer in adults [9,10]. UM is an aggressive cancer, and despite the successful treatment of the primary tumor (e.g., via plaque radiotherapy, proton beam radiotherapy, or enucleation), about half of the patients develop metastases, often hematogenously to the liver [9,10]. Therefore, a better understanding of UM biology and progression remains essential for the development of better prognostic biomarkers and new/adjuvant treatments. Currently, UM prognostication for metastatic potential relies on clinical information (i.e., the tumor size and location/extension), and when available, the tumor biopsy-based molecular information (i.e., from gene expression, chromosomal aberration, or gene mutation analysis) and/or histopathological information depending on obtainable tumor material (in vivo by fine-needle aspiration biopsy from the eyes undergoing eye-preserving therapy or ex vivo from the enucleated eyes). While the primary tumor biopsy-based molecular information has been shown to significantly improve UM prognostication [11,12,13,14,15,16], the liquid biopsy-based approaches (molecular analyses of body fluids), which are increasingly used in other cancers, have also increasingly become a focus of UM studies in recent years [2,17]. Liquid biopsy constitutes not only a less invasive alternative (to direct tumor biopsy) but also provides additional advantages such as repeat testing (to monitor therapy response, local recurrence, and metastatic potential) and the ability to capture the heterogeneous nature of cancer [2,18]. Given that UMs predominantly arise from the choroid in the posterior eye compartment (in >90% of cases) [9,10], the vitreous likely represents the most informative liquid biopsy source to study these choroidal melanomas (CMs), as it is expected to be enriched in tumor-derived molecules, because of its close proximity to these posteriorly located tumors [19,20].

Tumor microenvironment (TME), which includes both cellular (e.g., immune cells, fibroblasts, and endothelial cells) and soluble components, is a well-recognized player in tumor progression and metastasis. An inflammatory TME, involving macrophage and lymphocyte infiltration, has been linked to enhanced tumor growth/spread and poor prognosis in UM [14,21,22,23,24]. Inflammatory mediators and angiogenic factors constitute the key soluble components of TME [3,22,24,25,26]. These key soluble proteins (i.e., cytokines) were specifically and statistically investigated in the vitreous of UM-affected eyes in three previous studies, each involving ≥30 samples despite the rare availability of such collections [3,25,26]. In an initial study by Boyd et al. [25], elevated levels of VEGF-A were detected in the vitreous of enucleated eyes with UM (n = 30) compared to the aqueous humor of the eyes undergoing cataract surgery (n = 16). Later, two studies [3,26] were published examining multiple cytokines in the vitreous of enucleated eyes with UM using bead array technology. The study by Nagarkatti-Gude et al. [3] examined 27 cytokines in 33 UM-bearing eyes vs. 9 controls (eyes from the tissue bank, with no known ocular pathology) and reported 9 vitreous cytokines with altered levels (8 upregulated and 1 downregulated) in UM-bearing eyes. Likewise, the study by Dunavoelgyi et al. [26] evaluated 28 cytokines in 34 UM-affected eyes vs. 36 controls (eyes undergoing vitrectomy for vitreomacular traction with or without macular hole) and identified 11 vitreous cytokines significantly elevated in UM-affected eyes. Of the vitreous cytokines evaluated in at least two of these previous studies [3,25,26], several were consistently found upregulated in UM-affected eyes (i.e., IL-6, IL-8, IP-10, MCP-1, MIP-1α, and RANTES) whereas others yielded different results (i.e., IL-7, TNF-α, and VEGF). Of the vitreous cytokines examined in only one of these previous studies [3,26], those found dysregulated in UM-bearing eyes included Flt-3 ligand, IL-1, PDGF-AA, MIP-1β, and IL-1ra. Additionally, these previous UM studies have revealed correlations between the levels of several vitreous cytokines and the tumor size, which is a well-known prognostic factor in UM [11,12,27].

In this study, we used a bead-based multiplex immunoassay panel to evaluate 41 cytokines in vitreous samples obtained from 32 eyes, including 18 with CM (prior to plaque radiotherapy or following enucleation) and 14 controls (eyes undergoing vitrectomy for symptomatic vitreous floater, macular hole, or epiretinal membrane) in order (i) to seek replication of previous vitreous cytokine findings in an independent study sample, (ii) to investigate new cytokines included in this extended bead array panel for their relevance to CM, and (iii) to perform exploratory analyses within the CM group to identify potentially prognosis-relevant vitreous cytokines by investigating their associations with different prognostic categories (as defined by tumor biopsy-based gene expression profiling (GEP) [28]) as well as their correlations with the tumor dimensions.

## 2. Materials and Methods

A total of 32 subjects were included in the study, which was approved by the University of Michigan Institutional Review Board and adhered to the tenets of the Declaration of Helsinki.

The cases comprised 18 CM patients (mean age ± SD: 61.2 ± 18.9 years, 50% male) who underwent plaque radiotherapy (n = 12) or enucleation (n = 6) at the Kellogg Eye Center, University of Michigan. Demographic and clinical features of CM patients are presented in Table 1. Primary tumor biopsy (for GEP-based prognostication) and vitreous samples were obtained in the operating room at the time of radioactive plaque placement or enucleation procedure (prior to plaque radiotherapy or following enucleation). Vitreous samples were collected before the primary tumor biopsy. Under sterile conditions, a caliper was used to mark 4.0 mm and 3.5 mm posterior to the limbus for phakic and pseudophakic eyes, respectively, over the quadrant of conjunctiva away from the tumor. A 25-gauge × 5/8-inch needle was introduced through the marked site into the mid-vitreous cavity. A maximum of 0.1 mL of vitreous fluid was gently aspirated into a 1 mL syringe. Vitreous samples were immediately frozen and stored at −80 °C until analysis. No complications were observed as related to vitreous aspiration/sampling. Tumor biopsy was performed using a transscleral (n = 15) or transvitreal (n = 3) approach, and the obtained samples were stored at −80 °C until shipped in dry ice to the CAP-accredited and CLIA-certified laboratory that performs the GEP-based molecular classification (DecisionDx-UM^®^, Castle Biosciences, Phoenix, AZ, USA). DecisionDx-UM is a clinically validated and commercially available 15-gene assay, which stratifies UMs into two major metastatic risk groups [28]: Class 1 with low risk and Class 2 with high risk. Of 18 CM cases included in this study, 11 were classified as having Class 1 tumors and 7 as having Class 2 tumors. We used this GEP class information as a surrogate for the metastatic potential to evaluate the prognostically relevant vitreous cytokines in CM, given that not all patients were followed-up long enough to clinically assess the metastatic outcome.

The controls comprised 14 non-CM patients (mean age ± SD: 69 ± 9 years, 43% male), whose vitreous samples were collected during the vitrectomy procedure that was performed to treat other posterior eye conditions with only negligible pathological alterations [1], which included epiretinal membrane (n = 7), macular hole (n = 6), and symptomatic vitreous floater (n = 1) [5].

The vitreous levels of 41 cytokines were measured using a bead-based multiplex immunoassay panel (EMD Millipore, Darmstadt, Germany) following the manufacturer’s instructions [5]. Cytokines included in this immunoassay kit were EGF, FGF-2, Eotaxin/CCL11, TGF-α, G-CSF, Flt-3L, GM-CSF, Fractalkine/CX3CL1, IFN-α2, IFN-γ, GRO/CXCL1, IL-10, MCP-3/CCL7, IL-12p40, MDC/ADAM11, IL-12p70, PDGF-AA, IL-13, PDGF-AB/BB, IL-15, sCD40L, IL-17A, IL-1RA, IL-1α, IL-9, IL-1β, IL-2, IL-3, IL-4, IL-5, IL-6, IL-7, IL-8, IP-10/CXCL10, MCP-1/CCL2, MIP-1α/CCL3, MIP-1β/CCL4, RANTES/CCL5, TNF-α, TNF-β, and VEGF (full names are provided in Tables S1 and S2). Of these, eight cytokines (TGF-α, Fractalkine/CX3CL1, IFN-α2, GRO/CXCL1, MCP-3/CCL7, MDC/ADAM11, sCD40L, and TNF-β) were newly evaluated as part of a bead array-based vitreous cytokine analysis of UM [3,26].

Each sample was assessed in triplicate using 25 µL of vitreous. Quality controls included in each plate/run were utilized for batch normalization, when applicable, using a multiplication factor of the global mean over the batch mean. Median fluorescence intensity (MFI) values were extracted for each cytokine, and the average of technical replicates was used as a relative quantification of the cytokine abundance. In the methodological papers published by Breen et al. [29,30] on multiplexed immunoassays, the advantages of using MFI values over calculated absolute concentration values are well documented for low abundant analytes and include the following: no missing values (because all signals are retained with no out-of-range concerns), no need to specify concentration detection limits (hence preventing loss of information and related false conclusions), greater statistical power and discriminating ability (hence reducing the likelihood of missing true associations), and higher reproducibility (because high variability is usually caused by the use of standards to estimate concentrations, not by raw fluorescence intensities) [29,30]. The MFI-based analysis is therefore a better choice when multiplexed immunoassays are used to study the body fluids that yield multiple low signals, such as the ocular fluids.

For statistical analysis, nonparametric tests were preferred over parametric tests, as done in previous studies [3,26], because of the non-normal distribution of data. Vitreous cytokine levels were compared among different study groups (CM vs. non-CM groups and Class 2 vs. Class 1 CM subgroups) using the Wilcoxon rank sum test. Natural log transformed values were utilized to create scatter plots for individual cytokines and to visualize the cytokine profile differences among different study groups using a heatmap or principal component analysis (PCA) plot. Correlations between the vitreous cytokine levels and tumor dimensions (thickness and largest basal diameter (LBD)) were assessed using the Spearman’s rank correlation. All analyses were conducted using the R project for statistical computing (https://www.r-project.org/, last accessed on 10 July 2023). In CM vs. non-CM analysis (replication of previous findings or assessment of new cytokines included in the extended bead array panel), the *p*-values were adjusted (adj. *p*) for multiple comparisons with a controlled false discovery rate by implementing the widely used Benjamini and Hochberg method [31]. Our CM case-only analysis was exploratory in nature, where the nominal significance (*p* < 0.05) was used to identify and nominate the candidate cytokines for follow-up in future studies.

## 3. Results

**Figure 1:**
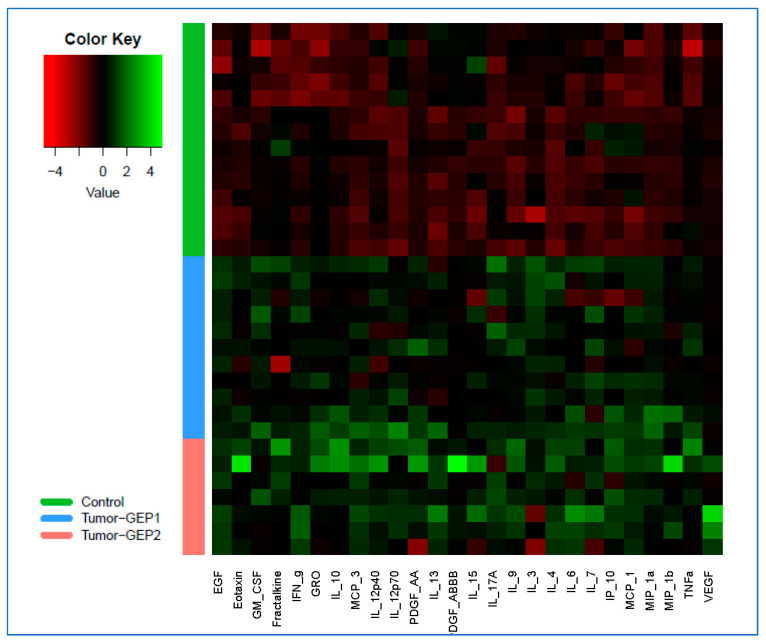
Heatmap illustrating the vitreous profiles of twenty-six cytokines differentially expressed in choroidal melanoma (CM)-bearing eyes compared to CM-free control eyes. Green indicates increased expression while red indicates reduced expression. The tumor group included 18 CM patients, of which 11 were classified as having Class 1—low risk (GEP1) and 7 as having Class 2—high risk (GEP2) tumors based on the primary tumor biopsy-based gene expression profiling (GEP) test used for metastatic risk prediction (DecisionDx-UM^®^) [28]. The control group included 14 non-CM patients. Vitreous cytokine levels are based on the MFI (median fluorescence intensity) values that were extracted/used for relative quantification of the cytokine abundance (see Methods) and are displayed here on a logarithmic scale (see Table S1 for the full names of the cytokines and details on individual cytokine analysis results).

A total of twenty-six cytokines (MIP-1α/CCL3, EGF, IL-9, IFN-γ, IL-4, IL-10, MCP-3/CCL7, PDGF-AA, VEGF, GM-CSF, MCP-1/CCL2, MIP-1β/CCL4, IL-12p40, TNF-α, IL-17A, Eotaxin/CCL11, IL-6, IL-3, IL-12p70, IP-10/CXCL10, IL-13, IL-7, GRO/CXCL1, Fractalkine/CX3CL1, PDGF-AB/BB, and IL-15) were found to be significantly upregulated in the vitreous of CM-affected eyes compared to non-CM (control) eyes (Table S1, Figure S1). These associations remained significant also after correcting for multiple comparisons (adj. *p* < 0.05). The differences in vitreous profiles of these 26 cytokines among the CM-affected and non-CM (control) eyes are illustrated in a heatmap displaying the cytokine levels on a logarithmic scale (Figure 1). Of these CM-associated cytokines, three (MCP-3/CCL7, GRO/CXCL1, and Fractalkine/CX3CL1) were among those newly investigated (not included in previous studies [3,26]) as part of a multiplex bead array-based vitreous cytokine analysis of UM.

**Figure 2:**
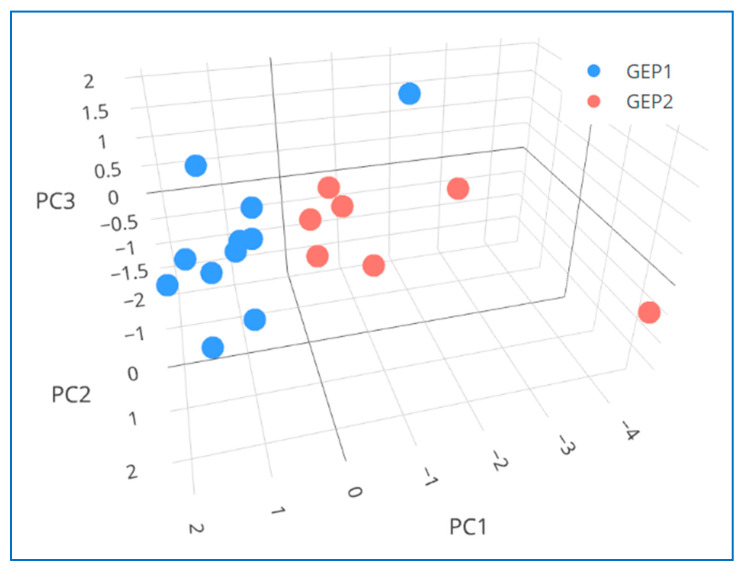
Three-dimensional plot illustrating the principal component analysis (PCA) results for six vitreous cytokines differentially expressed in eyes with choroidal melanomas (CMs) of different prognostic classes. CM group included 18 patients, of which 11 were classified as having Class 1—low risk (GEP1) and 7 as having Class 2—high risk (GEP2) tumors based on the primary tumor biopsy-based gene expression profiling (GEP) test used for metastatic risk prediction (DecisionDx-UM^®^) [28]. Vitreous cytokine levels are based on the MFI (median fluorescence intensity) values that were extracted/used for relative quantification of the cytokine abundance (see Methods) and are displayed here on a logarithmic scale. In PCA, the first three PCs were found to cumulatively explain 77% of data variance (PC1: 51%, PC2: 15%, and PC3: 11%), and the clustering of eighteen vitreous samples based on the expression profiles of six prognostically relevant cytokines (MCP-3/CCL7, PDGF-AB/BB, TNF-β, G-CSF, IL-13, and IL-3) has indicated a potential separation between different CM prognostic classes: all but one (10 of 11) GEP1 samples clustered together and separately from the GEP2 samples.

Within the CM group, our exploratory analysis detected five vitreous cytokines (PDGF-AB/BB, G-CSF, MCP-3/CCL7, IL-13, and TNF-β) in relatively higher and one cytokine (IL-3) in relatively lower levels in eyes with GEP Class 2—high risk vs. Class 1—low risk tumors (*p* < 0.05) (Table S2, Figure S2). The assessment of these six cytokines’ vitreous profiles using PCA has revealed a potential separation between the CM subgroups with high or low metastatic risk (Figure 2). Furthermore, our exploratory analysis identified three additional vitreous cytokines (TGF-α, IL-4, and VEGF) with a trend toward elevated levels in eyes with Class 2 vs. Class 1 tumors (Table S2, Figure S2). Interestingly, all these nine vitreous cytokines, which showed a potential (or a trend toward a potential) to distinguish the CM-affected eyes in different GEP-based prognostic categories, were also among those that showed correlation(s) with the tumor dimension(s) (*p* < 0.05) (see the next paragraph and Table 2). Moreover, the observed associations/correlations appeared to be in the same direction regarding the metastatic risk, such that those upregulated in eyes with high-risk Class 2 tumors were also positively correlated with the tumor dimension(s) whereas those downregulated in eyes with Class 2 tumors were also negatively correlated with the tumor dimension(s). Of these nine potentially CM prognosis-relevant cytokines, three (MCP-3/CCL7, TNF-β, and TGF-α) were among those newly investigated as part of a bead array-based vitreous cytokine analysis of UM.

Overall, our analysis has revealed multiple tumor size-associated vitreous cytokines, the levels of which correlated with one or more of the tumor dimensions (thickness and/or largest basal diameter (LBD)) (*p* < 0.05) (Table 2, Figures S3 and S4). These included twelve cytokines (IP-10/CXCL10, Eotaxin/CCL11, TNF-β, IL-6, IL-12p40, PDGF-AB/BB, MCP-3/CCL7, IL-2, VEGF, Fractalkine/CX3CL1, IL-4, and G-CSF) positively correlated with both tumor thickness and LBD, nine cytokines (PDGF-AA, sCD40L, IL-10, MDC/ADAM11, GRO/CXCL1, TGF-α, IL-8, IL-9, and IL-13) positively correlated with the tumor thickness, one cytokine (MCP-1/CCL2) positively correlated with the tumor LBD, and one cytokine (IL-3) negatively correlated with the tumor thickness (Table 2, Figures S3 and S4). Of these tumor size-associated cytokines, seven (TNF-β, MCP-3/CCL7, Fractalkine/CX3CL1, GRO/CXCL1, sCD40L, TGF-α, and MDC/ADAM11) were among those newly investigated as part of a bead array-based vitreous cytokine analysis of UM.

## 4. Discussion

The important role of chronic inflammation and angiogenesis in tumor pathogenesis and progression is now well recognized. These biological processes are mediated by interactions among various cellular and soluble components of the TME, ultimately leading to either a pro-tumorigenic (growth and spread) or anti-tumorigenic (regression) outcome in UM [3,21,22,23,24,25,26]. Given the growing interest in using less invasive and more advantageous liquid biopsy-based approaches to study and manage solid cancers, the protein markers (including inflammatory mediators and angiogenic factors) have also been increasingly investigated in ocular fluids of UM-affected eyes in recent years [3,4,20,25,26,32,33,34,35,36,37,38,39]. While both aqueous and vitreous humor have been used for this purpose (obtained in vivo or ex vivo from the eyes undergoing eye-preserving therapy or enucleation surgery), the data on vitreous remain limited due to fewer studies that focused on this ocular fluid by statistically analyzing either a selected set of cytokines (<30 analytes) [3,25,26] or a small discovery cohort (<10 UM samples) for a large number of proteins [20]. Additional studies of the vitreous in UM are therefore warranted, especially given that the majority of these melanomas arise from the choroid (CMs) [9,10], and the vitreous represents the most adjacent (and likely the most informative) ocular fluid to study [19,20] to better understand these mainly posteriorly located tumors.

In the present study, we examined the vitreous samples obtained from 32 eyes (18 with CM and 14 controls) for an extended set of selected cytokines (n = 41), using a multiplex bead array technology and an MFI-based analytical approach (more optimal for ocular fluids—see Methods), with the goals of identifying the cytokines associated with CM presence, and/or potentially relevant to CM prognosis (using primary tumor GEP-based prognostication as a surrogate for the metastatic potential) and/or correlating with the tumor dimensions. In brief, our study results replicated several previously reported CM-associated vitreous cytokines, implicated some new/additional ones as relevant to CM pathogenesis, and identified a candidate set of prognostically relevant vitreous cytokines with potential to distinguish the CMs with high or low metastatic risk.

### 4.1. Vitreous Cytokines Dysregulated in CM-Bearing Eyes Compared to CM-Free Control Eyes

By analyzing the vitreous samples obtained predominantly in vivo from CM-affected and control eyes, we identified multiple cytokines significantly upregulated in CM-bearing eyes (Figure 1 and Figure S1). These included six cytokines (MCP-1/CCL2, MIP-1α/CCL3, MIP-1β/CCL4, IP-10/CXCL10, IL-6, and PDGF-AA) that were similarly found in elevated levels in UM-bearing eyes in previous vitreous cytokine studies [3,26]. Of these cytokines, five (MCP-1/CCL2, MIP-1α/CCL3, IP-10/CXCL10, IL-6, and PDGF-AA) were also among those found upregulated in the aqueous humor of UM-affected eyes in one or more previous studies [26,33,34,35], further supporting their important roles in UM pathogenesis. MCP-1/CCL2 is associated with increased recruitment of pro-tumorigenic macrophages to TME in multiple cancers [40] and is similarly believed to play an important role in immune cell trafficking to the tumor site in UM [23,41]. Two other chemotactic cytokines, MIP-1α/CCL3 and MIP-1β/CCL4, are ligands for the chemokine receptor CCR5, which upon stimulation leads to several pro-tumorigenic effects including immunosuppression, angiogenesis, and metabolic reprogramming [42]. IP-10/CXCL10 is another chemotactic cytokine, which binds to CXCR3, and its expression by macrophages was shown to correlate with T cell infiltration and increased recruitment of immunosuppressive regulatory T cells to TME in UM [3,43]. IL-6 is a major proinflammatory cytokine that can mediate tumor–host interactions through multiple mechanisms including the recruitment and activation of macrophages, deactivation of macrophage tumor cytotoxicity, effects on other immune cells, and promotion of angiogenesis [44,45]. Also involved in the regulation of angiogenesis, the PDGF signaling pathway is well known for its role in cell growth and survival [46]. In summary, the current knowledge on the functions of the cytokines consistently found upregulated in the vitreous of UM-bearing eyes in several (current and previous [3,26]) studies further emphasizes the critical role of tumor growth-promoting inflammatory and angiogenic microenvironment in UM pathogenesis.

In addition, three chemotactic cytokines (MCP-3/CCL7, Fractalkine/CX3CL1, and GRO/CXCL1), which were among those newly investigated in this study (not included in previous bead array-based vitreous cytokine analyses of UM [3,26]), were also found in significantly elevated levels in the vitreous of CM-affected eyes. MCP-3/CCL7 is known to attract/activate leukocytes and regulate angiogenesis [47,48]. In a previous study that examined MCP-3/CCL7 in the aqueous humor of UM-containing eyes, the level of this cytokine showed correlation with the density of tumor-infiltrating CD68^+^ macrophages [34]. Fractalkine/CX3CL1 is similarly involved in the regulation of tumor-associated macrophage infiltration/polarization and the angiogenesis [49,50]. Likewise, GRO/CXCL1 can promote tumor growth and angiogenesis and regulate UM invasion [46,51]. While the functional relevance of these newly implicated vitreous cytokines is strongly supported by the published studies, our new findings are considered provisional until replicated/confirmed in additional UM studies.

### 4.2. Vitreous Cytokines Differentially Expressed in Eyes with CMs of Different Prognostic Classes and/or Correlated with the Tumor Dimensions

Accumulating evidence strongly links tumor angiogenesis and immunological microenvironment (development of an inflammatory phenotype) to genetic evolution of UM [52,53,54,55]. The genetic predictors of poor prognosis in UM include specific genetic events (monosomy 3, 8q gain, BAP1 loss) and changes in gene expression profile (Class 2 GEP) [52,53,54,55]. In this study, primary tumor biopsy-based GEP information [28] was utilized as a surrogate for the metastatic potential to investigate the prognostically relevant vitreous cytokines, which in turn may serve as liquid biopsy-based molecular biomarkers for CM. Unlike the genetic events (which alone do not provide functional information), the GEP and cytokine analyses can provide (at the RNA and protein level, respectively) a functional snapshot of the sum effects of tumor genetics, epigenetic changes, and host–tumor interactions (e.g., immune response) [56,57,58,59].

When we compared vitreous cytokine levels between different primary tumor GEP-defined prognostic classes (high-risk Class 2 vs. low-risk Class 1) in our CM group, we identified six differentially expressed cytokines (MCP-3/CCL7, PDGF-AB/BB, TNF-β, G-CSF, IL-13, and IL-3) (Figure S2). In previous cancer studies [26,46,60,61,62,63,64], these cytokines have been individually linked to the regulation of tumor growth, survival, angiogenesis, and/or escape from the immune response. In the PCA plot generated using the vitreous expression profiles of these six cytokines, all but one (10 of 11) Class 1 samples clustered together and separately from the Class 2 samples (Figure 2). Given that up to ~10% of Class 1 tumors may still metastasize despite their overall better prognosis compared to Class 2 tumors [16], it would be interesting to determine whether that single Class 1 case that landed more closely to the Class 2 cluster in PCA (Figure 2) will eventually develop metastasis in long-term follow-up. Interestingly, all these GEP class-associated cytokines (PDGF-AB/BB, MCP-3/CCL7, TNF-β, G-CSF, IL-13, and IL-3) were also among the tumor size-correlated cytokines (Table 2). This provides further support for the prognostic relevance of these cytokines, consistent with the previous observations that both the tumor size and GEP-based classification are among the major prognosticators for UM [11,12,65]. Moreover, of these six CM prognosis-relevant cytokines, four (PDGF-AB/BB, MCP-3/CCL7, IL-13, and IL-3) were also among the vitreous cytokines significantly dysregulated in CM-affected eyes compared to non-CM (control) eyes (Figure 1 and Figure S1).

In previous UM studies [3,26], no association was detected between vitreous cytokine levels and prognostic tumor chromosome 3 status; however, chromosomal abnormalities are expected to only partially correlate with prognostic tumor GEP status [55], as the latter provides a functional snapshot of both the tumor and its microenvironment influenced by both genetic and epigenetic factors [56,57,58,59]. In a recent vitreous study of UM [20], a large number of proteins (n = 1000) was evaluated in relation to GEP classes in a discovery sample comprising 8 UM subjects, and even though the 20 proteins selected for follow-up in 11 UM subjects did not include our cytokines of interest, the study results similarly suggested the prognostic potential of vitreous protein profiling. In two recent UM studies, which evaluated aqueous humor proteins in relation to GEP classes (for 1469 proteins) [36] or as related to clinicopathological and cytogenetic prognosticators (for 84 proteins) [38], the study results similarly suggested the potential of aqueous humor protein profiling to differentiate between different prognostic categories. Aqueous humor molecular profiles appear to be primarily influenced by the ciliary body involvement in UM, probably because of the close anatomic relationship between the aqueous humor and the ciliary body [18,38]. Notably, MCP-3/CCL7 was among the most prognostically significant proteins identified in one of these aqueous humor studies of UM [38], and together with our findings, this cytokine emerges as a candidate that could serve as a prognostic biomarker in both ocular fluids and for both anteriorly and posteriorly located tumors.

Using the six prognostically relevant cytokines identified in our study as the focus molecules, we checked the Ingenuity Knowledge Base of IPA (QIAGEN, Redwood City, CA, USA) for related biological networks and directly interacting molecules in human primary cells and tissues based on the data curated from published/public resources. Our search has revealed a total of three networks, a merged version of which is shown in Figure S5. Notably, in addition to the six focus cytokines that differed between the GEP classes (MCP-3/CCL7, PDGF-AB/BB, TNF-β, G-CSF, IL-13, and IL-3), two of the three cytokines that showed a trend for such difference (IL-4 and VEGF) also ended up in the same IPA networks (Figures S2 and S5). Similar to a recent vitreous study of UM [20], our search has revealed the STAT3 pathway as a major signaling pathway involved in UM prognosis (Figure S5). The identified IPA networks have also captured three molecules (SERPINE1, TNFRSF1A, and TNFRSF1B) previously linked to GEP class differences in a recent aqueous humor study of UM [36], as well as a molecule (SATB1) encoded by one of the 12 discriminating genes targeted in UM GEP test (DecisionDx-UM [28,66]) (Figure S5). All these observations provide compelling support for the biological significance of the prognostically relevant cytokines and networks identified in our exploratory study.

Apart from the cytokines found to be associated with both the GEP class and tumor size, our study identified several other tumor size-associated vitreous cytokines, the levels of which correlated with the tumor thickness and/or LBD (Table 2). Of the vitreous cytokines previously reported to be associated with UM size parameters [3,26], those consistently replicated in all—current and previous—studies include IP-10/CXCL10, IL-6, IL-8, PDGF-AA, and G-CSF. Compared to these previous studies, our study identified a larger number of tumor size-associated vitreous cytokines, probably due to several factors: (i) unlike the previous studies that examined only enucleated eyes with large tumors, our study included both plaque-treated and enucleated eyes (a collection less skewed toward large tumors), which might have facilitated our detection of additional cytokines correlated with tumor dimensions; (ii) our study used a bead array with an extended cytokine panel and several newly analyzed cytokines were among those found significantly correlated with the tumor size; and (iii) our study employed an MFI-based analytical approach (see Methods), which is proven to be more optimal [29,30] for the body fluids that contain low abundant cytokines such as the ocular fluids.

### 4.3. Study Strengths and Limitations

While our study provides valuable information for future follow-up studies, it has both strengths and limitations. The strengths of our study include the majority of vitreous samples being collected in vivo (from 12 of 18 CM cases and all 14 CM-free controls), the analysis of a CM population with a broader tumor size distribution (from small to large), the availability of primary tumor biopsy-based GEP data for prognostic classification as a surrogate for metastasis, and the use of an MFI-based multiplex immunoassays analysis approach (more optimal for ocular fluids which often yield multiple low signals). The limitations include the impossibility of collecting in vivo samples from healthy eyes (thus the use of CM-free surrogate controls) and the relatively small sample size (due to both the rarity of CMs and lack of large vitreous collections), and thus the exploratory nature of our CM case-only analyses. Nevertheless, our study has uncovered several UM-relevant vitreous cytokines worthy of follow-up in larger studies to confirm their candidacy for liquid biopsy-based biomarker development and/or new therapeutic targeting.

## 5. Conclusions and Future Directions

Despite the increasing popularity of liquid biopsy-based approaches in the cancer field, there is currently no such approach established for UM to address the issues associated with primary tumor biopsies (e.g., risk for complications, tumor heterogeneity, non-repeatability for follow-up, and inapplicability to small tumors). While the studies of ocular fluids (anteriorly located aqueous humor and posteriorly located vitreous humor) in UM are still limited in number and often small in size due to the rarity of this cancer, they increasingly reveal candidate molecules as potential therapeutic targets and/or prognostic biomarkers.

Our analysis of vitreous cytokines in CM (the predominant form of UM occurring in the posterior eye compartment) has consistently replicated several previously reported UM-associated vitreous cytokines (MCP-1/CCL2, MIP-1α/CCL3, MIP-1β/CCL4, IP-10/CXCL10, IL-6, and PDGF-AA), which represent potential therapeutic targets. Three of these cytokines (IP-10/CXCL10, IL-6, and PDGF-AA) were also among those consistently associated with the tumor dimensions in all—current and previous—vitreous studies of UM. Moreover, our exploratory analysis in the CM group has revealed several prognostically relevant (GEP class-associated and tumor size-correlated) vitreous cytokines (MCP-3/CCL7, PDGF-AB/BB, TNF-β, G-CSF, IL-13, and IL-3), which represent not only additional therapeutic targets but also potential liquid biopsy-based prognostic biomarkers. Four of these cytokines (MCP-3/CCL7, PDGF-AB/BB, IL-13, and IL-3) showed significance in all analyses performed in our study (CMs vs. controls, GEP Class 2 vs. Class 1 CMs, and correlations with tumor dimensions). Of these, MCP-3/CCL7 is of special interest as it was also found to be prognostically significant in a recent aqueous humor study of UM [38]. The UM prognosis-relevant proteins consistently detected in both vitreous and aqueous humor studies may represent the best biomarker candidates as being informative about all UMs, regardless of their (posterior or anterior) location.

Given that blood sampling is a minimally invasive procedure, future testing of these potential biomarkers in plasma/serum may also have a significant value; however, because UM patients are usually of advanced age and often present with various comorbidities (that may also influence blood molecular profiles), ocular fluids may prove to be more informative about UM-specific biomarkers. Going forward, a simultaneous and longitudinal assessment of both ocular and systemic fluids would constitute the best strategy for the identification/selection of the most sensitive, specific, and feasible protein biomarkers for UM. Because of their critical roles in shaping host immune response and regulating tumor growth and spread, cytokines hold great promise as new therapeutic targets for improving the clinical outcomes in UM and other cancers.

## Figures and Tables

**Table 1:** Demographic and clinical features of choroidal melanoma (CM) patients (n = 18).

Age (Mean ± SD)	61.2 ± 18.9
Gender (n)	
Male	9
Female	9
Quadrant location (n)	
Temporal	3
Nasal	4
Inferior	4
Superior	4
Posterior pole	3
Antero-posterior location (n)	
Anterior to the equator	6
Posterior to the equator	8
Posterior pole	3
Posterior pole to CB	1
Ciliary body (CB) involvement ^1^ (n)	
Yes	5
No	13
Largest basal diameter (LBD) ^2^ (n)	
<12 mm	2
≥12 mm	16
Tumor thickness ^3^ (n)	
<7 mm	12
≥7 mm	6
Subretinal fluid (n)	
Yes	15
No	3
Drusen (n)	
Yes	5
No	13
Treatment (n)	
Plaque radiotherapy	12
Enucleation	6
Gene expression profiling class ^4^ (n)	
Class 1	11
Class 2	7

^1^ CB involvement was determined by clinical examination and ocular transillumination and confirmed by B-scan ultrasonography and ultrasound biomicroscopy. ^2^ LBD was estimated by indirect ophthalmoscopy with fundus mapping and ocular transillumination (when possible). Mean LBD was 15 mm (median 14.5 mm, range 9 to 24 mm). ^3^ Tumor thickness was measured using B-scan ultrasonography by placing the markers between the top of the tumor just under the retina and the base of the tumor just above the sclera. Mean tumor thickness was 5.9 mm (median 5.2 mm, range 1 to 10.8 mm). ^4^ Gene expression profiling of the primary tumor was performed using a clinically validated and commercially available 15-gene assay (DecisionDx-UM^®^) [28] to predict the metastatic risk: Class 1—low risk and Class 2—high risk.

**Table 2:** Correlations of vitreous cytokine measures with the tumor dimensions.

Cytokine ^1^	Largest Basal Diameter		Tumor Thickness	
	Spearman Correlation	*p*-Value ^2^	Spearman Correlation	*p*-Value ^2^
Interferon gamma-induced protein-10 (IP-10/CXCL10) *	0.59	**0.0094**	0.75	**0.0003**
Eotaxin (CCL11) *	0.66	**0.0027**	0.74	**0.0005**
Tumor necrosis factor beta (TNF-β) **†**	0.79	**0.0001**	0.70	**0.0012**
Interleukin-6 (IL-6) *	0.65	**0.0032**	0.67	**0.0022**
Platelet-derived growth factor A (PDGF-AA) *	0.43	0.0752	0.67	**0.0022**
Interleukin-12 (IL-12p40) *	0.76	**0.0002**	0.67	**0.0024**
Platelet-derived growth factor B (PDGF-AB/BB) *^,^†	0.59	**0.0096**	0.66	**0.0027**
Monocyte chemoattractant protein 3 (MCP-3/CCL7) *^,^†	0.67	**0.0022**	0.63	**0.0047**
Interleukin-2 (IL-2)	0.52	**0.0286**	0.62	**0.0056**
Soluble CD40 ligand (sCD40L)	0.45	0.0602	0.61	**0.0071**
Vascular endothelial growth factor (VEGF) *	0.49	**0.0397**	0.61	**0.0078**
Interleukin-10 (IL-10) *	0.45	0.0624	0.60	**0.0088**
Macrophage-derived chemokine (MDC/ADAM11)	0.39	0.1120	0.60	**0.0092**
Growth-regulated alpha protein (GRO/CXCL1) *	0.37	0.1278	0.59	**0.0097**
Interleukin-3 (IL-3) *^,^†	−0.29	0.2405	−0.57	**0.0144**
Transforming growth factor-alpha (TGF-α)	0.39	0.1114	0.54	**0.0195**
Interleukin-8 (IL-8)	0.37	0.1349	0.54	**0.0213**
Fractalkine (CX3CL1) *	0.58	**0.0111**	0.54	**0.0221**
Interleukin-9 (IL-9) *	0.41	0.0898	0.51	**0.0294**
Interleukin-4 (IL-4) *	0.66	**0.0030**	0.49	**0.0409**
Interleukin-13 (IL-13) *^,^†	0.35	0.1588	0.49	**0.0411**
Granulocyte colony-stimulating factor (G-CSF) **†**	0.50	**0.0360**	0.47	**0.0467**
Monocyte chemoattractant protein 1 (MCP-1/CCL2) *	0.51	**0.0294**	0.45	0.0622
Macrophage inflammatory protein 1-beta (MIP-1β/CCL4) *	0.45	0.0636	0.43	0.0722
Interferon gamma (IFN-γ) *	0.44	0.0654	0.41	0.0903
Fibroblast growth factor-2 (FGF-2)	0.39	0.1066	0.36	0.1366
Interleukin-5 (IL-5)	0.39	0.1104	0.36	0.1442
Tumor necrosis factor alpha (TNF-α) *	0.11	0.6628	0.35	0.1595
Regulated upon Activation, Normal T cell Expressed, and Secreted (RANTES/CCL5)	0.13	0.6043	0.31	0.2169
Interleukin-15 (IL-15) *	0.28	0.2665	0.30	0.2224
Granulocyte-macrophage colony-stimulating factor (GM-CSF) *	−0.18	0.4667	−0.29	0.2426
Macrophage inflammatory protein 1-alpha (MIP-1α/CCL3) *	0.26	0.3062	0.24	0.3427
Fms-like tyrosine kinase-3 ligand (Flt-3L)	−0.02	0.9503	0.18	0.4731
Epidermal growth factor (EGF) *	0.38	0.1227	0.17	0.4927
Interleukin-7 (IL-7) *	−0.22	0.3837	−0.13	0.6040
Interleukin-1 alpha (IL-1α)	0.04	0.8727	0.11	0.6563
Interleukin-1 receptor antagonist protein (IL-1RA)	−0.11	0.6524	−0.11	0.6685
Interleukin-1 beta (IL-1β)	0.07	0.7719	0.09	0.7194
Interleukin-12 (IL-12p70) *	−0.01	0.9551	0.06	0.8160
Interferon alpha-2 (IFN-α2)	−0.17	0.4918	−0.06	0.8260
Interleukin-17A (IL-17A) *	0.08	0.7592	−0.01	0.9724

^1^ The cytokines that showed significance in CM vs. non-CM group comparison are marked with (*) while those that showed difference in GEP Class 2 vs. Class 1 CM subgroup comparison are marked with (**†**). ^2^ The *p*-values were determined by Spearman’s rank correlation and are shown as rounded to four decimal places. Cytokines are ordered based on corresponding *p*-values (first for the tumor thickness then for the largest basal diameter), and the *p*-values of <0.05 are highlighted in **bold**.

## Data Availability

All the data reported are presented here (in the article or Supplementary Materials).

## Supplementary Materials

The following supporting information can be downloaded at: https://www.mdpi.com/article/10.3390/cancers15143701/s1, Table S1: Comparison of vitreous cytokine measures between choroidal melanoma (CM) and non-CM (control) groups; Table S2: Comparison of vitreous cytokine measures between different tumor GEP-defined prognostic classes/groups; Figure S1: Scatter plots of vitreous cytokine measures and the comparison between the choroidal melanoma (CM) and non-CM control groups; Figure S2: Scatter plots of vitreous cytokine measures and the comparison between different prognostic classes within the choroidal melanoma (CM) group; Figure S3: Scatter plots showing the correlations between vitreous cytokine measures and the tumor thickness within the choroidal melanoma (CM) group; Figure S4: Scatter plots showing the correlations between vitreous cytokine measures and the largest tumor basal diameter within the choroidal melanoma (CM) group; Figure S5: A merged version of three biological networks captured by a search in the Ingenuity Knowledge Base of IPA (QIAGEN) for the molecules directly interacting with the vitreous cytokines differentially expressed in eyes with choroidal melanomas (CMs) of different prognostic classes.
