# Supplementary material for: Investigating Vitreous Cytokines in Choroidal Melanoma
Source: Cancers (Basel). 2023 Jul 21;15(14):3701. doi: 10.3390/cancers15143701 (PMC10378009; doi:10.3390/cancers15143701)
Supplement: Supplementary file 1 [file cancers-15-03701-s001.zip › cancers-2442556-supplementary.pdf]

Table S1. Comparison of vitreous cytokine measures between choroidal melanoma (CM) and non-CM (control) groups.

Cytokine[1]	Melanoma (n=18)	Control (n=14)	Comparison
	Median (IQR)	Median (IQR)	p-Value[2]
Macrophage inflammatory protein 1-alpha (MIP-1α/CCL3)	70.96 (58.60-82.09)	22.58 (18.59-24.45)	<0.0001
Epidermal growth factor (EGF)	28.18 (27.61-30.38)	19.31 (17.23-20.23)	<0.0001
Interleukin-9 (IL-9)	17.51 (16.40-18.96)	13.14 (11.78-13.98)	<0.0001
Interferon gamma (IFN-γ)	18.48 (17.01-19.71)	13.82 (12.19-14.47)	<0.0001
Interleukin-4 (IL-4)	19.52 (18.25-20.20)	14.51 (14.12-16.55)	<0.0001
Interleukin-10 (IL-10)	21.80 (20.33-25.64)	16.98 (16.02-17.52)	<0.0001
Monocyte chemoattractant protein 3 (MCP-3/CCL7)	12.71 (11.48-13.37)	8.19 (7.80-8.55)	<0.0001
Platelet-derived growth factor A (PDGF-AA)	2587.72 (1559.41-4863.03)	1047.09 (978.15-1119.50)	<0.0001
Vascular endothelial growth factor (VEGF)	17.63 (15.81-35.14)	12.46 (11.29-13.72)	<0.0001
Granulocyte-macrophage colony-stimulating factor (GM-CSF)	24.77 (23.95-26.42)	21.19 (19.53-21.65)	<0.0001
Monocyte chemoattractant protein 1 (MCP-1/CCL2)	21725.98 (19970.48-22728.88)	12873.77 (11476.59-16450.78)	<0.0001
Macrophage inflammatory protein 1-beta (MIP-1β/CCL4)	27.03 (24.13-35.82)	17.77 (14.96-20.44)	<0.0001
Interleukin-12 (IL-12p40)	16.84 (16.28-18.32)	13.45 (12.60-14.39)	<0.0001
Tumor necrosis factor alpha (TNF-α)	30.08 (28.53-32.44)	24.91 (19.71-25.56)	<0.0001
Eotaxin (CCL11)	25.80 (22.34-29.95)	18.09 (16.48-20.24)	<0.0001
Interleukin-17A (IL-17A)	15.24 (14.33-16.19)	12.71 (12.43-13.12)	<0.0001
Interleukin-6 (IL-6)	244.77 (108.99-426.93)	45.21 (35.59-63.36)	0.0001
Interleukin-3 (IL-3)	36.27 (33.20-40.42)	24.08 (23.03-25.51)	0.0002
Interleukin-12 (IL-12p70)	13.92 (12.74-15.10)	10.34 (9.97-12.45)	0.0003
Interferon gamma-induced protein-10 (IP-10/CXCL10)	4107.64 (1724.99-10444.72)	561.20 (382.85-862.85)	0.0003
Interleukin-13 (IL-13)	12.43 (11.07-14.26)	9.56 (8.60-11.18)	0.0006
Interleukin-7 (IL-7)	44.33 (31.10-54.94)	20.66 (18.60-25.39)	0.0008
Growth-regulated alpha protein (GRO/CXCL1)	79.62 (65.95-130.50)	62.24 (24.72-69.26)	0.0041
Fractalkine (CX3CL1)	14.25 (12.94-16.75)	12.50 (9.75-13.28)	0.0175
Platelet-derived growth factor B (PDGF-AB/BB)	16.14 (13.53-19.52)	8.28 (7.02-18.84)	0.0262
Interleukin-15 (IL-15)	72.54 (61.88-90.82)	56.32 (43.43-70.76)	0.0275
Fms-like tyrosine kinase-3 ligand (Flt-3L)	36.49 (17.58-63.44)	19.86 (16.90-24.75)	0.1064
Fibroblast growth factor-2 (FGF-2)	13.09 (11.68-15.28)	10.81 (8.67-13.09)	0.1149
Interleukin-1 alpha (IL-1α)	38.14 (35.94-41.86)	26.29 (23.08-64.99)	0.1239
Interferon alpha-2 (IFN-α2)	13.63 (12.18-15.18)	14.93 (14.41-15.78)	0.1435

Granulocyte colony-stimulating factor (G-CSF)	15.36 (12.43-20.96)	13.12 (10.77-14.95)	0.1542
Transforming growth factor-alpha (TGF-α)	28.52 (21.73-35.61)	25.07 (17.14-29.10)	0.1543
Interleukin-2 (IL-2)	12.37 (11.39-13.76)	10.44 (9.67-24.47)	0.2464
Interleukin-8 (IL-8)	249.65 (101.46-574.06)	133.70 (105.60-178.13)	0.2666
Tumor necrosis factor beta (TNF-β)	9.97 (9.49-10.91)	10.42 (9.23-29.51)	0.2960
Soluble CD40 ligand (sCD40L)	9.90 (9.25-10.77)	8.29 (7.64-110.77)	0.3518
Interleukin-5 (IL-5)	8.77 (8.07-9.65)	7.74 (6.95-12.93)	0.3716
Interleukin-1 receptor antagonist protein (IL-1RA)	26.10 (15.26-34.12)	15.36 (10.90-90.91)	0.4361
Interleukin-1 beta (IL-1β)	10.71 (9.48-11.55)	10.87 (8.90-19.39)	0.6347
Macrophage-derived chemokine (MDC/ADAM11)	15.86 (8.71-69.74)	15.20 (11.64-21.95)	0.7757
Regulated upon Activation, Normal T cell Expressed, and Secreted (RANTES/CCL5)	13.98 (12.43-20.76)	16.50 (9.63-17.19)	0.8344

[1]Vitreous cytokine levels are based on the MFI (median fluorescence intensity) values that were extracted/used for relative quantification of the cytokine abundance (see Methods); IQR, interquartile range.
[2]The *p*-values were determined by the Wilcoxon rank sum test and are shown as rounded to four decimal places. Cytokines are ordered based on corresponding *p*-values. All observed associations remained significant also after correcting for multiple comparisons using the Benjamini-Hochberg method (adj. $P < 0.05$).

Table S2. Comparison of vitreous cytokine measures between different tumor GEP-defined prognostic classes/groups.[1]

Cytokine[2]	Class 1 (n=11)	Class 2 (n=7)	Comparison
	Median (IQR)	Median (IQR)	*p*-Value[3]
Platelet-derived growth factor B (PDGF-AB/BB)	12.00 (11.59-13.92)	22.50 (16.66-24.09)	0.0075
Granulocyte colony-stimulating factor (G-CSF)	13.67 (13.16-17.00)	23.67 (17.59-30.91)	0.0098
Interleukin-3 (IL-3)	45.83 (42.25-50.66)	38.67 (25.58-39.84)	0.0127
Monocyte chemoattractant protein 3 (MCP-3/CCL7)	12.67 (10.67-12.92)	13.67 (13.09-14.83)	0.0161
Interleukin-13 (IL-13)	12.67 (12.09-14.59)	16.33 (14.59-16.33)	0.0232
Tumor necrosis factor beta (TNF-β)	13.67 (12.66-14.09)	15.67 (14.50-16.16)	0.0459
Transforming growth factor-alpha (TGF-α)	24.83 (22.75-32.17)	44.00 (33.17-54.17)	0.0514
Interleukin-4 (IL-4)	20.67 (19.42-21.33)	22.00 (21.34-23.00)	0.0565
Vascular endothelial growth factor (VEGF)	15.00 (14.16-16.58)	83.33 (20.25-342.00)	0.0571
Granulocyte-macrophage colony-stimulating factor (GM-CSF)	27.33 (26.34-30.25)	26.00 (23.66-26.50)	0.0772
Fractalkine (CX3CL1)	12.50 (11.59-13.75)	15.50 (12.41-17.16)	0.1029
Epidermal growth factor (EGF)	24.83 (23.91-25.59)	27.17 (25.58-27.34)	0.1227
Interferon gamma-induced protein-10 (IP-10/CXCL10)	2168.00 (1754.00-5928.50)	9995.00 (5352.50-16651.00)	0.1259
Interleukin-1 alpha (IL-1α)	48.33 (45.16-54.42)	45.50 (42.42-47.66)	0.1469
Interleukin-6 (IL-6)	169.83 (122.83-394.75)	468.00 (362.75-611.92)	0.1471

Soluble CD40 ligand (sCD40L)	14.33 (13.00-15.00)	15.33 (14.34-16.33)	0.1882
Interleukin-9 (IL-9)	17.17 (16.59-18.08)	19.33 (17.25-21.33)	0.2381
Interleukin-12 (IL-12p40)	17.50 (16.84-18.91)	17.67 (17.50-19.59)	0.2764
Interferon gamma (IFN-γ)	18.33 (17.33-19.16)	19.17 (18.09-21.92)	0.2971
Monocyte chemoattractant protein 1 (MCP-1/CCL2)	24917.00 (21116.50-26612.50)	26322.00 (24926.00-26783.00)	0.3283
Macrophage inflammatory protein 1-alpha (MIP-1α/CCL3)	75.00 (66.08-81.42)	58.83 (54.66-87.09)	0.3283
Interleukin-2 (IL-2)	16.00 (15.09-18.09)	18.00 (15.34-21.41)	0.3644
Eotaxin (CCL11)	25.83 (23.50-32.83)	30.00 (26.25-38.50)	0.3649
Macrophage inflammatory protein 1-beta (MIP-1β/CCL4)	31.50 (26.25-36.34)	31.33 (30.00-57.58)	0.4409
Interleukin-17A (IL-17A)	16.67 (15.67-17.41)	17.33 (16.50-17.84)	0.4963
Platelet-derived growth factor A (PDGF-AA)	2300.00 (1538.50-4206.00)	2679.00 (1925.50-6299.50)	0.5360
Interleukin-12 (IL-12p70)	14.50 (13.66-15.59)	15.67 (13.83-16.09)	0.5857
Interleukin-15 (IL-15)	89.50 (80.59-107.33)	102.67 (81.84-160.58)	0.5962
Interleukin-10 (IL-10)	22.67 (20.41-25.00)	21.67 (21.59-35.34)	0.6179
Fms-like tyrosine kinase-3 ligand (Flt-3L)	45.00 (29.91-77.00)	50.50 (12.16-75.66)	0.6590
Growth-regulated alpha protein (GRO/CXCL1)	30.67 (26.75-50.16)	35.83 (28.42-63.08)	0.6590
Interleukin-1 receptor antagonist protein (IL-1RA)	42.17 (24.59-48.00)	32.67 (16.59-236.25)	0.6590
Interferon alpha-2 (IFN-α2)	18.17 (16.58-19.75)	19.50 (16.66-21.50)	0.6834
Tumor necrosis factor alpha (TNF-α)	33.33 (32.25-35.42)	32.83 (30.59-37.08)	0.6834
Fibroblast growth factor-2 (FGF-2)	13.17 (11.59-15.09)	13.83 (12.16-15.25)	0.7169
Interleukin-7 (IL-7)	50.33 (31.25-64.00)	49.33 (35.92-54.34)	0.7242
Interleukin-8 (IL-8)	259.83 (125.09-533.66)	471.50 (117.25-1075.66)	0.7242
Interleukin-1 beta (IL-1β)	12.67 (11.84-15.00)	13.83 (13.67-14.66)	0.7508
Interleukin-5 (IL-5)	9.67 (8.92-10.25)	8.83 (8.67-10.25)	0.8558
Regulated upon Activation, Normal T cell Expressed, and Secreted (RANTES/CCL5)	13.67 (12.50-18.67)	14.50 (11.25-45.75)	0.9278
Macrophage-derived chemokine (MDC/ADAM11)	20.67 (15.25-65.16)	17.83 (10.34-133.00)	0.9639

[1]Gene expression profiling (GEP) of the primary tumor was performed using a clinically validated and commercially available 15-gene assay (DecisionDx- UM®) [28] to predict the metastatic risk: Class 1-low risk and Class 2-high risk.
[2]Vitreous cytokine levels are based on the MFI (median fluorescence intensity) values that were extracted/used for relative quantification of the cytokine abundance (see Methods); IQR, interquartile range.
[3]The *p*-values were determined by the Wilcoxon rank sum test and are shown as rounded to four decimal places. Cytokines are ordered based on corresponding *p*-values.

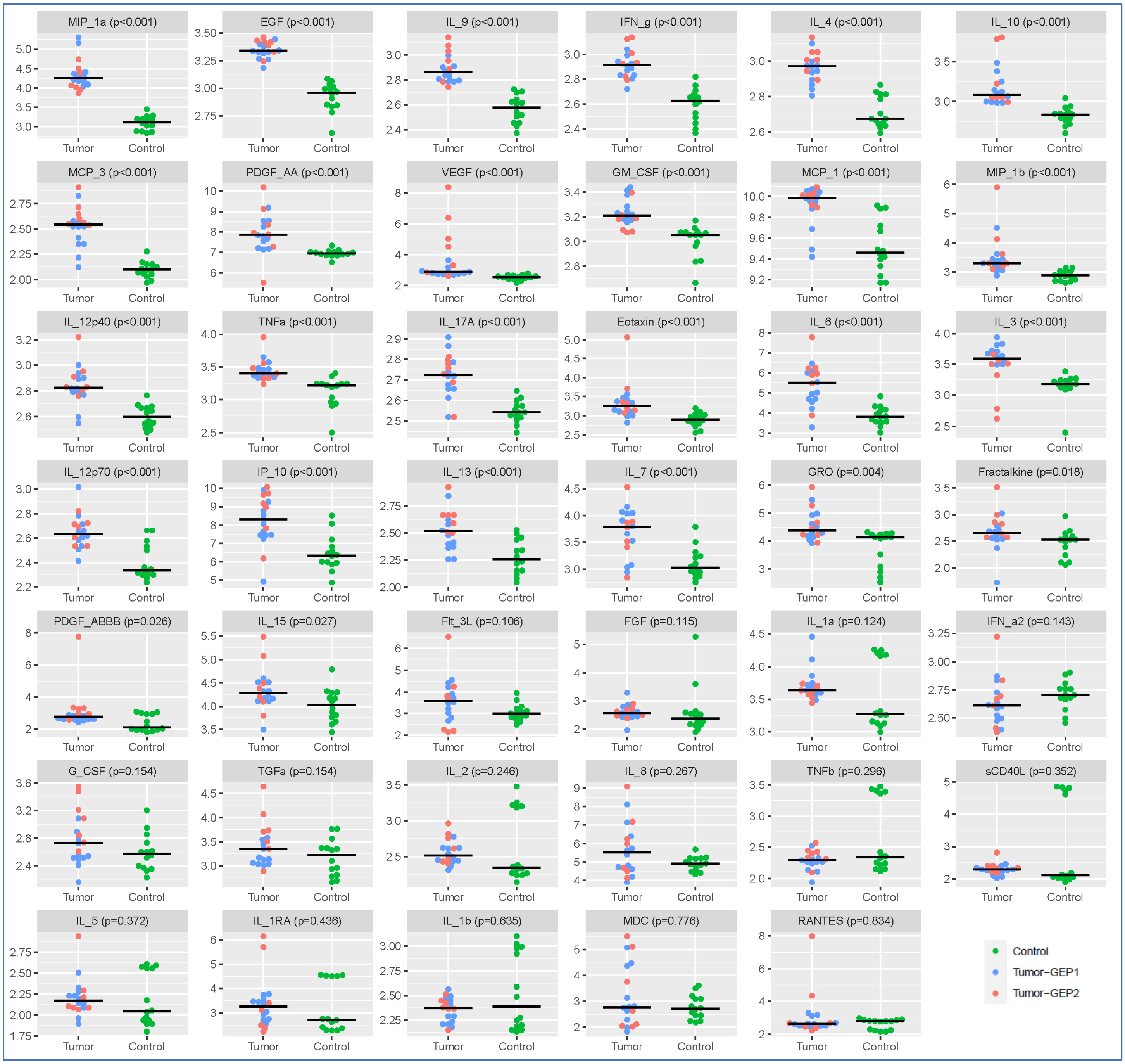

Figure S1. Scatter plots of vitreous cytokine measures and the comparison between the choroidal melanoma (CM) and non-CM control groups. The tumor group included 18 CM patients, of which 11 were classified as having Class 1-low risk (GEP1) and 7 as having Class 2-high risk (GEP2) tumors based on the primary tumor biopsy-based gene expression profiling (GEP) test used for metastatic risk prediction (DecisionDx-UM®) [28]. The control group included 14 non-CM patients. Vitreous cytokine levels are based on the MFI (median fluorescence intensity) values that were extracted/used for relative quantification of the cytokine abundance (see Methods) and are displayed here on a logarithmic scale. Horizontal lines are used to indicate the median in each group. The *p*-values were determined by the Wilcoxon rank sum test and are shown as rounded to three decimal places. Cytokines are ordered based on corresponding *p*-values, from the most to least significant (see **Table S1** for the full names of the cytokines and additional details on CM vs. non-CM group comparison results).

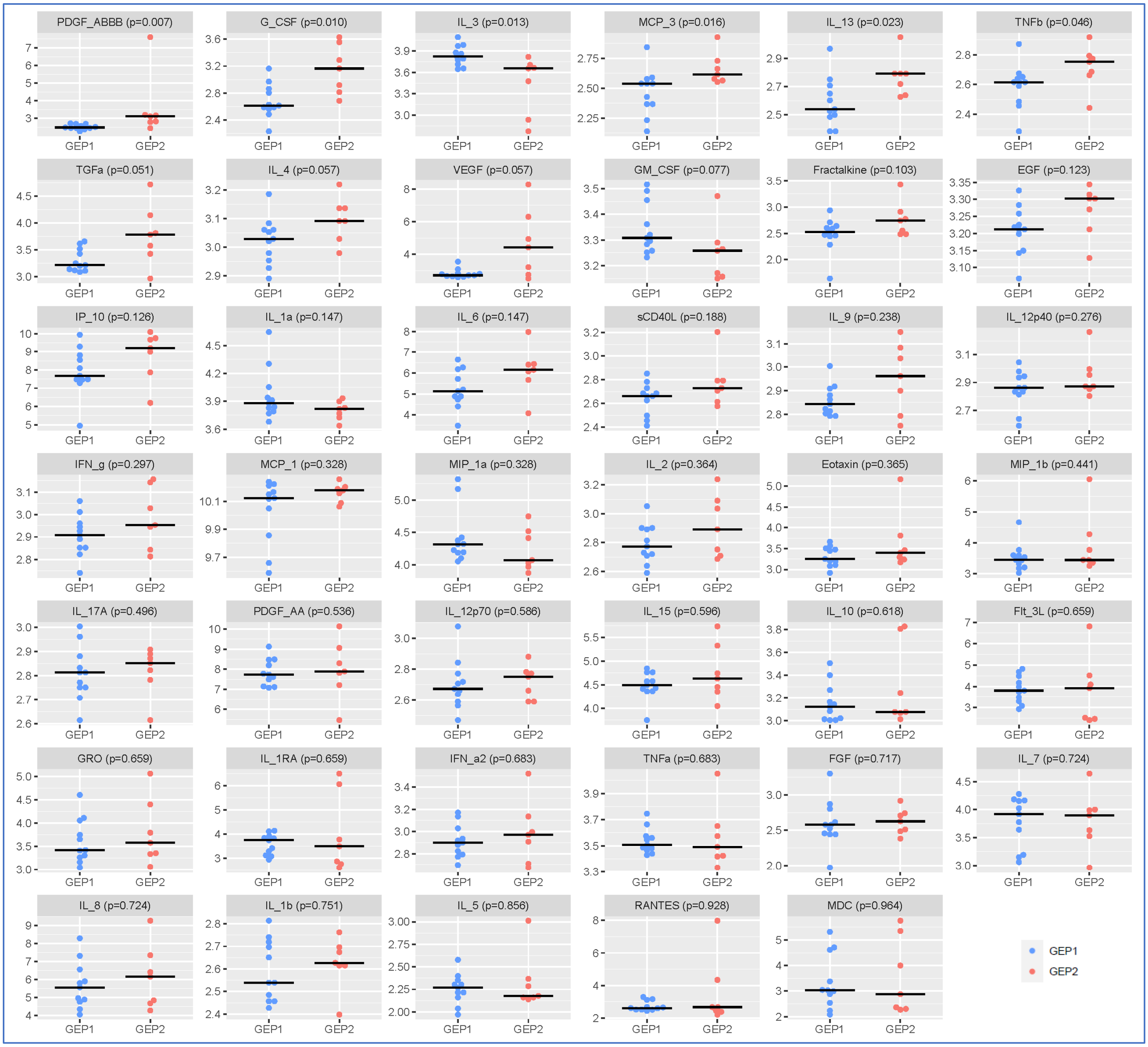

Figure S2. Scatter plots of vitreous cytokine measures and the comparison between different prognostic classes within the choroidal melanoma (CM) group. CM group included 18 patients, of which 11 were classified as having Class 1-low risk (GEP1) and 7 as having Class 2-high risk (GEP2) tumors based on the primary tumor biopsy-based gene expression profiling (GEP) test used for metastatic risk prediction (DecisionDx-UM®) [28]. Vitreous cytokine levels are based on the MFI (median fluorescence intensity) values that were extracted/used for relative quantification of the cytokine abundance (see Methods) and are displayed here on a logarithmic scale. Horizontal lines are used to indicate the median in each subgroup. The *p*-values were determined by the Wilcoxon rank sum test and are shown as rounded to three decimal places. Cytokines are ordered based on corresponding *p*-values, from the most to least significant (see **Table S2** for the full names of the cytokines and additional details on GEP Class 2 vs. Class 1 CM subgroup comparison results).

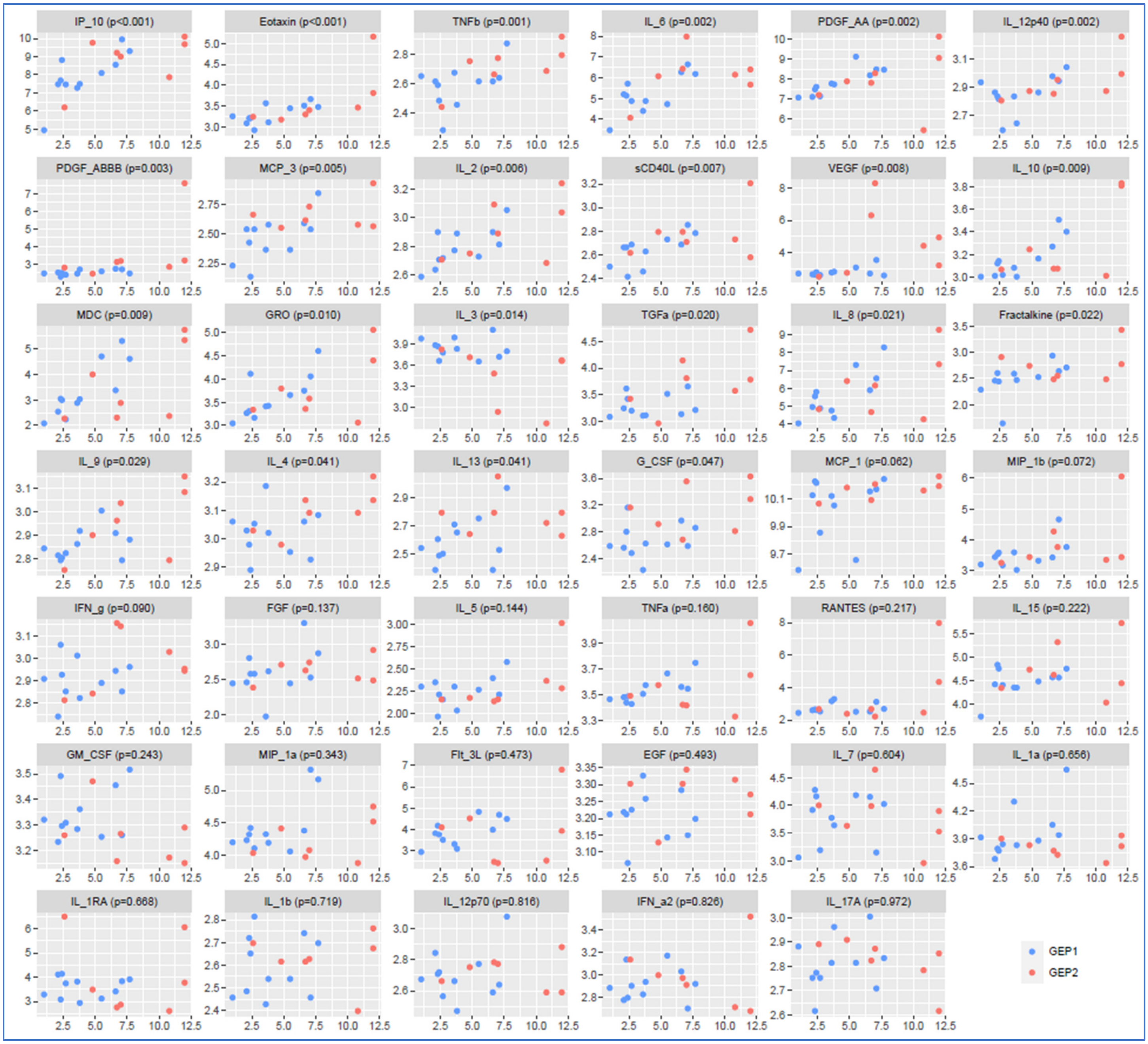

Figure S3. Scatter plots showing the correlations between vitreous cytokine measures and the tumor thickness within the choroidal melanoma (CM) group. CM group included 18 patients, of which 11 were classified as having Class 1-low risk (GEP1) and 7 as having Class 2-high risk (GEP2) tumors based on the primary tumor biopsy-based gene expression profiling (GEP) test used for metastatic risk prediction (DecisionDx-UM®) [28]. Vitreous cytokine levels are based on the MFI (median fluorescence intensity) values that were extracted/used for relative quantification of the cytokine abundance (see Methods) and are displayed here on a logarithmic scale (Y-axis). The *p*-values were determined by Spearman's rank correlation and are shown as rounded to three decimal places. Cytokines are ordered based on corresponding *p*-values, from the most to least significant (see **Table 2** for the full names of the cytokines and additional details on correlations of vitreous cytokine measures with the tumor dimensions).

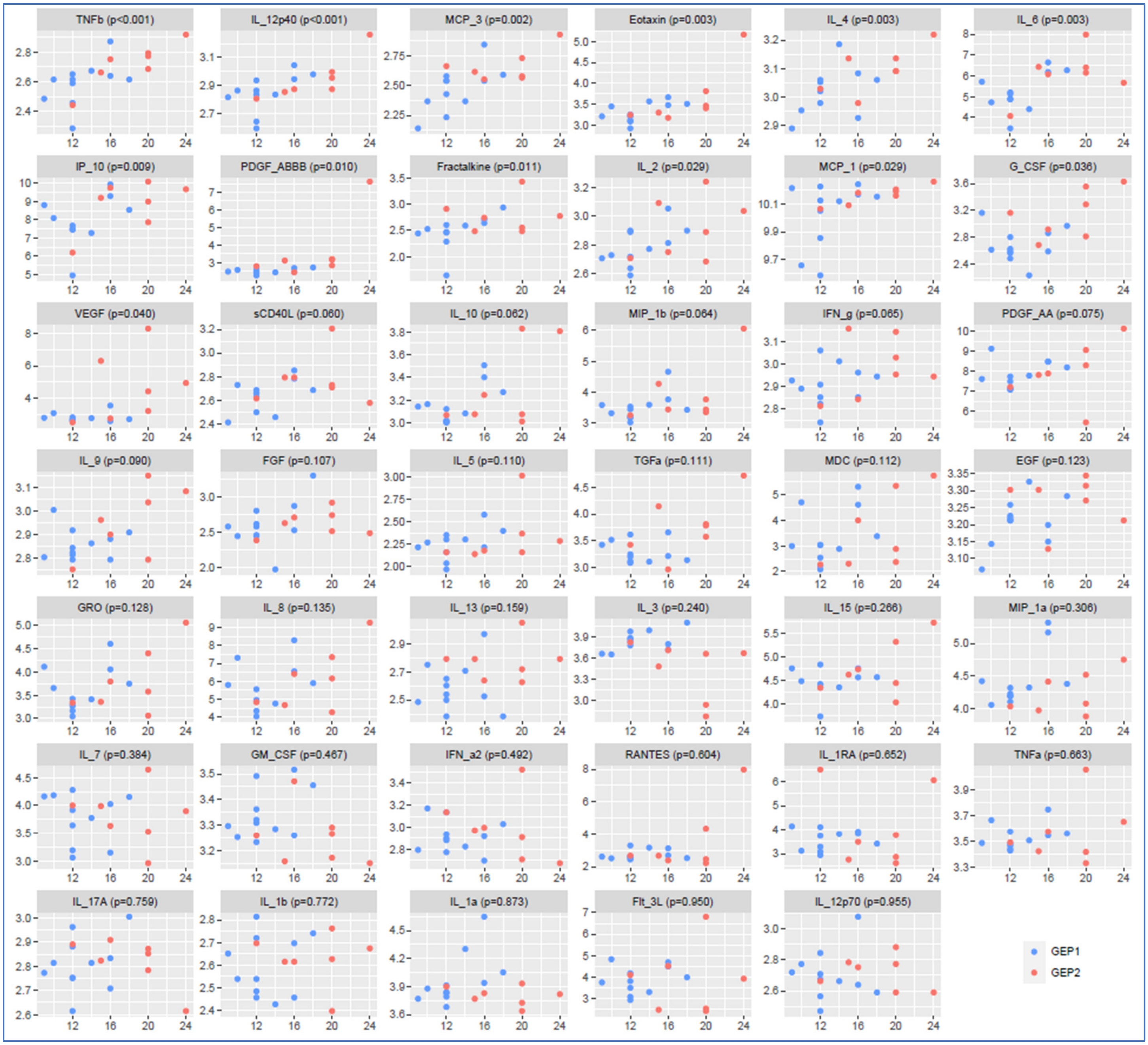

Figure S4. Scatter plots showing the correlations between vitreous cytokine measures and the largest tumor basal diameter within the choroidal melanoma (CM) group. CM group included 18 patients, of which 11 were classified as having Class 1-low risk (GEP1) and 7 as having Class 2-high risk (GEP2) tumors based on the primary tumor biopsy-based gene expression profiling (GEP) test used for metastatic risk prediction (DecisionDx-UM®) [28]. Vitreous cytokine levels are based on the MFI (median fluorescence intensity) values that were extracted/used for relative quantification of the cytokine abundance (see Methods) and are displayed here on a logarithmic scale (Y-axis). The *p*-values were determined by Spearman's rank correlation and are shown as rounded to three decimal places. Cytokines are ordered based on corresponding *p*-values, from the most to least significant (see **Table 2** for the full names of the cytokines and additional details on correlations of vitreous cytokine measures with the tumor dimensions).

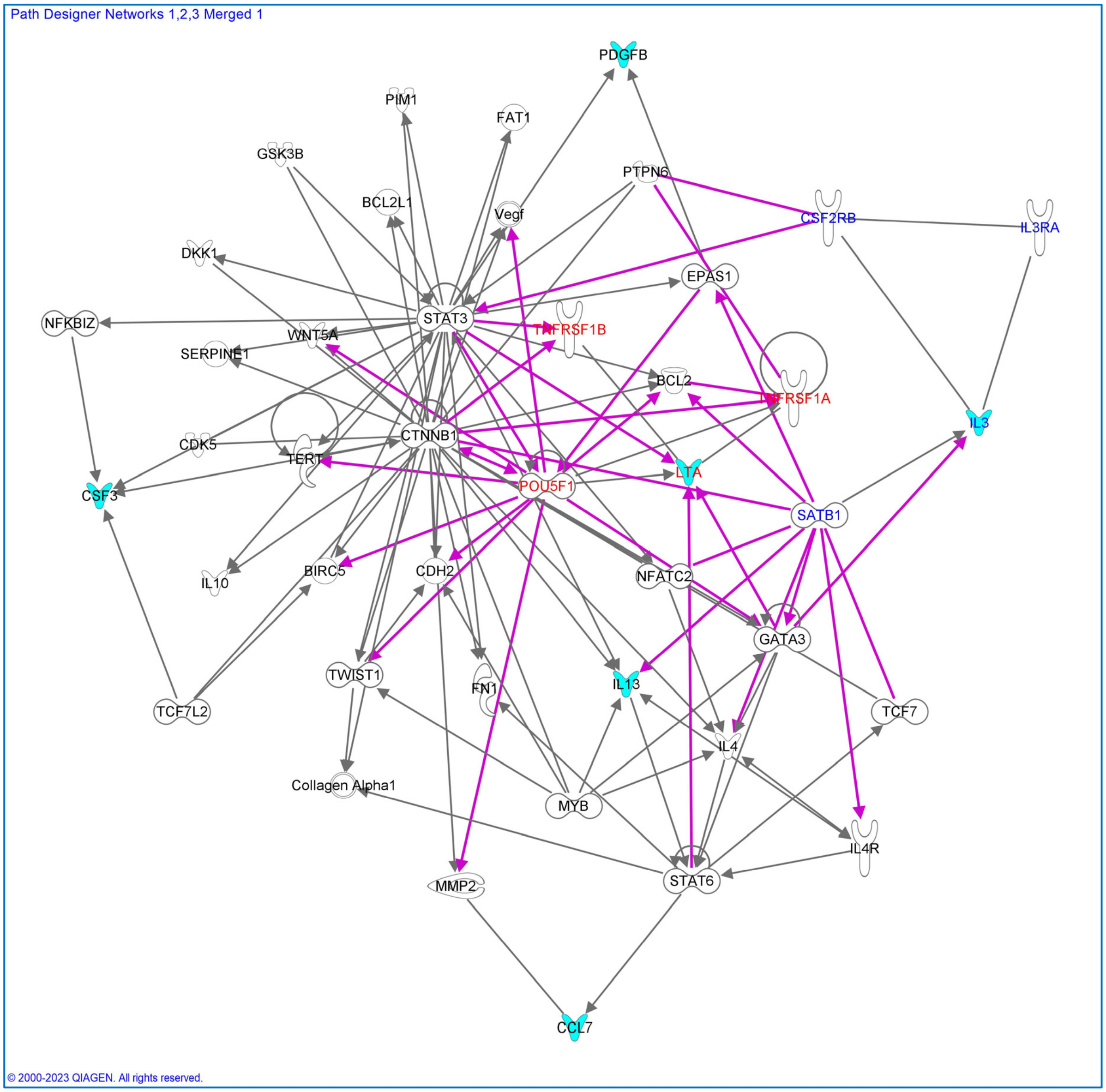

© 2000-2023 QIAGEN. All rights reserved.

Figure S5. A merged version of three biological networks captured by a search in the Ingenuity Knowledge Base of IPA (QIAGEN) for the molecules directly interacting with the vitreous cytokines differentially expressed in eyes with choroidal melanomas (CMs) of different prognostic classes (GEP1: low metastatic risk, GEP2: high metastatic risk). Using six prognostically relevant vitreous cytokines (MCP-3/CCL7, PDGF-AB/BB, TNF-β/LTA, G-CSF/CSF3, IL-13, IL-3) as the focus molecules (highlighted in blue) and the information available from human primary cells and tissues for direct interactions, a total of three IPA networks were captured: Network 1 comprising >30 molecules mainly interacting with STAT3 and/or CTNNB1 and including 4 focus cytokines (MCP-3/CCL7, PDGF-AB/BB, G-CSF/CSF3, IL-13) upregulated in the vitreous of GEP2 CMs, 2 vitreous cytokines (IL-4, VEGF) that showed trend for upregulation in GEP2 CMs, and one molecule (SERPINE1) previously shown upregulated in aqueous humor of GEP2 UMs [36]; Network 2 comprising 4 molecules including one focus cytokine (TNF-β/LTA) upregulated in the vitreous of GEP2 CMs and two molecules (TNFRSF1A, TNFRSF1B) previously shown upregulated in aqueous humor of GEP2 UMs [36]; Network 3 comprising 4 molecules including one focus cytokine (IL-3) downregulated in the vitreous of GEP2 CMs and a molecule (SATB1) encoded by one of 12 discriminating genes targeted in UM GEP test (one of 8 genes downregulated in GEP2 UMs) (DecisionDx-UM [28,66]). The gray lines indicate direct interactions within a Network whereas the purple lines indicate direct interactions between the Networks.
